# Biocatalytic Synthesis of Flavor Ester “Pentyl Valerate” Using *Candida rugosa* Lipase Immobilized in Microemulsion Based Organogels: Effect of Parameters and Reusability

**DOI:** 10.1155/2014/353845

**Published:** 2014-07-01

**Authors:** Tripti Raghavendra, Nilam Panchal, Jyoti Divecha, Amita Shah, Datta Madamwar

**Affiliations:** ^1^BRD School of Biosciences, Sardar Patel Maidan, Sardar Patel University, Satellite Campus, Vadtal Road, P.O. Box 39, Vallabh Vidyanagar, Gujarat 388120, India; ^2^Department of Statistics, Sardar Patel University, Vallabh Vidyanagar, Gujarat 388120, India

## Abstract

Pentyl valerate was synthesized biocatalytically using* Candida rugosa *lipase (CRL) immobilized in microemulsion based organogels (MBGs). The optimum conditions were found to be pH 7.0, temperature of 37°C, ratio of concentration of water to surfactant (Wo) of 60, and the surfactant sodium bis-2-(ethylhexyl)sulfosuccinate (AOT) for MBG preparation. Although kinetic studies revealed that the enzyme in free form had high affinity towards substrates (*K*_*m*_ = 23.2 mM for pentanol and 76.92 mM for valeric acid) whereas, after immobilization, the *K*_*m*_ values increased considerably (74.07 mM for pentanol and 83.3 mM for valeric acid) resulting in a slower reaction rate, the maximum conversion was much higher in case of immobilized enzyme (~99%) as compared to free enzyme (~19%). Simultaneous effects of important parameters were studied using response surface methodology (RSM) conjugated with Box-Behnken design (BBD) with five variables (process parameters), namely, enzyme concentration, initial water content (Wo), solvent used for MBG preparation, substrate ratio and time, and response as the final product formation, that is, pentyl valerate (%). The MBGs were reused for 10 consecutive cycles for ester synthesis. Efficacy of AOT/isooctane as dehydrating agent for extracting excess water from MBGs was found to exert a positive effect on the esterification reaction.

## 1. Introduction

With the emergence of “green chemistry” as an alternative to the conventional chemical methods, biocatalysis has gained a significant position in industrial biotechnology sector. A large number of enzymes have been produced and purified and they are replacing a number of chemical processes involved in production and processing of textiles, food and beverages, cosmetics, pharmaceuticals, and bulk and fine chemical synthesis [1–3]. Originally enzymes were studied and applied in media majorly consisting of aqueous phase as it constitutes their natural environment. It was soon realized that many enzymes behave as remarkable catalysts for synthetic reactions in nonaqueous medium [4]. It was very well explained and exemplified that enzymes function brilliantly in organic solvents in the presence/absence of added water [5]. Further, display of promiscuous activities by many enzymes (ability to catalyze reactions other than those catalyzed in their natural milieu) [6] has made this area of biocatalysis emerge as a whole new chapter pertaining to their structure and behavior in nonconventional media.

In particular, microbial lipases have proved to be one of the highest studied and applied enzymes in biotechnology [7]. Lipases (triacylglycerol acyl hydrolases, EC 3.1.1.3) are enzymes which catalyze the hydrolysis of oils and fats resulting in release of free fatty acids, diglycerides, monoglycerides, and glycerol in their natural milieu. In addition, they catalyze various other types of reactions resulting in a myriad of applications such as production of food supplements, flavors and aromas, cosmetics, pharmaceutical products, biodiesel, bulk and fine chemicals, and resolution of racemic compounds [8]. Intensive studies have been performed regarding properties and practical applications of lipases. For example, in their review, Mendes et al. have described the important features of porcine pancreatic lipase (PPL) in terms of its structure, properties, and immobilization techniques employed for PPL and its biotechnological applications in detail [9].

Although lipases (and other enzymes) function with exceptional efficiency in non-aqueous media, the common drawbacks associated with their use are separation and reusability due to their soluble/powder nature, and solvent induced denaturation caused by change in structure and flexibility of enzyme molecules owing to replacement of bound water molecules in the enzyme molecule by solvent, hence distorting its structure [10]. These problems have been very well tackled by immobilizing the enzymes using various techniques into/onto various ways of support [11] which not only aid in easy separation but also have been shown to enhance their activities in many cases [12]. One of the numerous methods for enzyme immobilization is microemulsion based organogels (MBGs). Also known as “double immobilization” [13], these systems are wonderfully designed to work under harsh nonaqueous conditions as they contain an aqueous core (for solubilizing enzymes and water soluble substrates), a surfactant interface (for surface active enzymes), and a nonaqueous continuous phase (for hydrophobic substrates). The gelling agents (such as gelatin), which help in solidification of the reverse micellar system and hence easy separation, also provide the second line of protection. The porous structure of MBGs allows diffusion of substrates and products across its cross-sectional area. Owing to all the advantages, this system of immobilization has been widely used for various applications [14].

One of the areas of lipase directed application is synthesis of short chain esters which are important constituents of flavoring agents. Such biocatalytically synthesized esters have an edge over their chemical counterparts as they excel in their flavor and are considered “natural” making them highly accepted [9, 15]. Pentyl valerate (amyl valerate or pentyl pentanoate) is one such ester that is used in dilute solutions to replicate the flavors of apple and sometimes pineapple. Present work was focused on synthesis of this ester using immobilized* Candida rugosa *lipase (CRL) in organic solvents. The important parameters affecting product formation have been studied individually and as interactive combinations. The MBGs were subjected to reusability studies and analyses by TGA and Karl-Fischer titration for estimation of water content after reusing.

## 2. Materials and Methods

### 2.1. Materials

Sodium bis-2-(ethylhexyl) sulfosuccinate (AOT), Tween 80, 1-hexanol (>98% purity, GC), 1-pentanol (>99% purity, GC), pentyl valerate (>98% purity, GC), and valeric acid (>98% purity, GC) were obtained from Fluka (Steinheim, Germany). Cetyl trimethyl ammonium bromide (CTAB) and Triton X-100 were obtained from Genei (Bangalore, India) and Hi-Media (Mumbai, India), respectively.* Candida rugosa *lipase (triacylglycerol acyl hydrolase, EC 3.1.1.3, type VII) with total activity of 1104 U/mg of solid and gelatin from porcine skin, type A (~300 Bloom) were procured from Sigma (Steinhein, Germany). All other organic solvents and chemicals used were of HPLC/GC grade and obtained from Spectrochem (Mumbai, India).

### 2.2. Methods

#### 2.2.1. Preparation of Reverse Micelles and MBGs Using Different Surfactants

Reverse micelles and MBGs were prepared as described in our previous study [16]. Reverse micelles were prepared using four surfactants, namely, AOT, CTAB, Triton X-100, and Tween 80 and solvents isooctane, n-hexane, and n-heptane in various combinations (Table S1 in Supplementary Material available online at http://dx.doi.org/10.1155/2014/353845). Appropriate amount of lipase (70 mg/mL) dissolved in buffer was added to the surfactant solution (100 mM) and mixed vigorously to obtain thermodynamically stable reverse micellar solution. In case of cationic and nonionic surfactants, 1-hexanol (cosurfactant) was added to stabilize the reverse micelles. Short chain alcohols are generally used in addition to surfactants to stabilize reverse micelles [14]. Optimum Wo and Po (in case of cationic and nonionic surfactants) were determined for each stable reverse micellar solution in the following manner:

(1) $$\begin{array}{c} \text{Wo}=\frac{\left[\text{water}\right]}{\left[\text{surfactant}\right]},\\ \text{Po}=\frac{\left[\text{co-surfactant}\right]}{\left[\text{surfactant}\right]}. \end{array}$$

Microemulsion based organogels (MBGs) were prepared by addition of molten gelatin (14%) maintained at 55°C to the reverse micelles (1 mL) and mixing vigorously till homogenous. The resulting mixture was cooled, poured into plastic Petri plates (diameter = 4.75 cm), and air-dried till the organogels exhibited constant weight. The dried gels (110 ± 10 *μ*m thickness) were cut into small pieces (0.5 cm × 0.5 cm) and used for esterification reaction.

#### 2.2.2. Esterification Reaction Conditions

The reaction mixture consisted of 20 mL of organic solvent containing 100 mM of each substrate (1-pentanol and valeric acid) in 250 mL glass stoppered flasks. To initiate the reaction, the enzyme was added to the reaction mixture and kept on orbital shaker at 37°C and 150 rpm.

*Effect of Immobilization and Reaction Parameters on Ester Synthesis*. Effects of the following immobilization and reaction parameters were assessed:

- surfactant for reverse micelle preparation: AOT, CTAB, Triton X-100, and Tween-80,
- pH: 5–8.8 of 100 mM strength (acetate buffer, pH 5, sodium phosphate buffer, pH 6, 7, and 8, and Tris-HCl buffer, pH 8.8),
- Wo: 10–100 (enzyme concentration of 5 mg/mL of reverse micelles),
- enzyme concentration: 10–100 mg/mL (Wo = 50, pH 7),
- organic solvent as reaction medium: isooctane, n-hexane, n-heptane, and cyclohexane,
- cyclic versus acyclic alkanes as reaction medium for ester synthesis,
- reaction temperature: 20–50°C.

#### 2.2.3. Study of Substrate Concentration on Reaction Kinetics

The effects of concentrations of valeric acid and pentanol on the initial rate of pentyl valerate synthesis were studied using free and immobilized lipase keeping the initial concentration of one of the substrates, that is, pentanol/valeric acid constant (100 mM), and varying the initial concentration of the other (50 mM, 75 mM, 100 mM, 125 mM, and 150 mM). One unit of enzyme activity was defined as amount of lipase required for synthesis of 1 *μ*mole of pentyl valerate in 1 minute at 37°C and pH 7. *K*_*m*_ and *V*_max⁡_ were determined for free and immobilized lipase using the Lineweaver-Burk equation. Consider

(2) $$\begin{array}{c} \frac{1}{v}=\;\frac{K_{m}}{\left[S\right]}\cdot \;\frac{1}{V_{\max}}+\;\frac{1}{V_{\max}}\;, \end{array}$$

where a plot of 1/*v*against 1/[*S*] gives a straight line with the slope *K*_*m*_/*V*_max⁡_ and intercepts on the *x*- and *y*- axis of −1/*K*_*m*_ and 1/*V*_max⁡_, respectively. *K*_*m*_ and *V*_max⁡_ were hence calculated by taking the reciprocals of 1/*K*_*m*_ and 1/*V*_max⁡_.

Where *v* is the reaction rate, *K*_*m*_ is the Michaelis constant, *S* is the substrate (valeric acid or pentanol), and *V*_max⁡_ is the maximum rate.

#### 2.2.4. Statistical Analysis

Study of the significant effects of various parameters and their interactions on esterification using response surface methodology (RSM) and Box-Behnken design (BBD) is considered. Studying combined effects of various parameters by conventional methods is time consuming and high number of experiments results in wastage of resources as well. Hence, employment of statistical tools such as response surface methodology (RSM) assists in a limited number of runs leading to predicted results followed by confirmatory experiments. In present work, RSM using BBD (Box-Behnken design) which entails full factorial search was employed for studying the simultaneous effects of five important process parameters chosen as independent variables, that is, Wo (*X*_1_), enzyme concentration (*X*_2_), substrate ratio (1-pentanol: valeric acid) (*X*_3_), time (*X*_4_), and solvent for MBG preparation (*X*_5_), on the response, namely, pentyl valerate yield. The specific ranges of the variables studied for their combinatorial effects on esterification percentage are shown in Table 1. Each of these independent variables was studied at three levels as per BBD in five variables with a total of 45 experimental runs. Sodium phosphate buffer (pH 7.0) for dissolving lipase, reaction temperature of 37°C, and cyclohexane as reaction medium were kept constant throughout the entire experimentation. The scheme of BBD in the coded levels of the variables is shown in Table 2.

For statistical calculations the independent variables were coded as

(3) $$\begin{array}{c} x_{i}=\frac{\left(X_{i}-X_{O}\right)}{{\delta X}_{i}}, \end{array}$$

where *X*_*i*_ represents the experimental value of variable; *X*_*O*_ is the midpoint of *X*_*i*_; *δX*_*i*_ indicates the step change in *X*_*i*_; and *x*_*i*_ is the coded value for *X*_*i*_, *i* = 1–5.

This response surface methodology allows creating a model of a second order equation that illustrates the response (in present work pentyl valerate yield) as a function of the five variables studied. Subsequently, pentyl valerate synthesis data was analyzed and response surface model given by the following equation was fitted. Consider

(4) $$\begin{array}{c} Y=\beta_{0}+\sum \beta_{i}x_{i}\;+\;\sum \beta_{ii}x_{i}^{2}\sum \beta_{ij}x_{ij}, \end{array}$$

where *β*_0_, *β*_*i*_, *β*_*ii*_, and *β*_*ij*_ symbolize the constant process effect, the linear effect of *X*_*i*_, quadratic effect of *X*_*i*_, and the interaction effect between *X*_*i*_ and *X*_*j*_, respectively, on pentyl valerate synthesis which is denoted by *Y*.

*Interpretation and Data Analysis*. The results of the experimental design were analyzed and interpreted using the MINITAB version 15 (PA, USA) statistical software. Also, the same software was applied for prediction of parameters yielding highest ester formation and creation of the curves describing the model and the process.

#### 2.2.5. Operational Stability of MBGs

After completion of one reaction cycle, the MBGs were separated from reaction mixture by filtration using Whatman filter paper and washed with cyclohexane 2-3 times for complete removal of residual substrates and product which might have diffused into/been adsorbed onto the organogel. They were then air-dried and reused for ester synthesis. After 3-4 cycles, when the ester production was observed to decrease considerably, the MBGs were treated with dehydrant solution (1 M AOT/isooctane) for 24 h for extraction of excess water (by-product of esterification reaction), given 2-3 solvent washes of neat isooctane for complete removal of AOT and used for next cycle of esterification.

### 2.3. Analytical Procedure

#### 2.3.1. Gas Chromatography

After initiation of the reaction, 100 *μ*L sample of the reaction mixture was withdrawn periodically every 24 h and immediately analyzed by gas chromatography (injection volume = 1 *μ*L) using Perkin Elmer, Model Clarus 500, USA, equipped with a flame ionization detector and RTX-20 (crossband 80% dimethyl-20% diphenyl polysiloxane) capillary column (30 m, 0.32 mmID, 1 *μ*m df), Restek, USA. The carrier gas was nitrogen at a split flow rate of 90 mL/min. The injector and detector temperatures were 250 and 280°C, respectively, and oven temperature was programmed to increase from 100 to 160°C at the rate of 20°C/min, from 160 to 165°C at the rate of 2°C/min, and from 165 to 175°C at the rate of 1°C/min. Ester identification and quantification were done by comparing the retention time and peak area of the sample with standard. Pure pentyl valerate (>98%, GC) was used as the external standard. 100 mM pentyl valerate was considered as 100% yield.

#### 2.3.2. Karl-Fischer Titration for Determination of Water Content

Water content of the three types or organogels (i) fresh MBGs (unused), (ii) MBGs reused for 8 cycles, and (iii) reused MBGs subjected to dehydrating agent (1 M AOT/isooctane) was determined by the Karl-Fischer method using Hydranal-E reagent by volumetric titration.

#### 2.3.3. Thermal Gravitometric Analysis

Dynamic thermogravimetric experiments were carried out using a Mettler Toledo (Switzerland) TGA/SDTA/821e thermal analyzer, allowing measurement of mass change. The system employed for this work was equipped with a PtRh furnace capable of operating from 25°C to 1500°C, the temperature being measured using type R thermocouple. The system is vacuum tight, allowing measurements to be conducted under controlled atmosphere. TGA-DSC analysis was performed on small samples (0.1–2.5 mg) and taken in alumina crucible without lid, in argon atmosphere (flow rate of argon gas, 200 mL/min). The temperature range was varied from room temperature to 300°C and with heating rate of 20°C/min. For isothermal analysis, the temperature was maintained at 100°C for 30 min.

## 3. Results and Discussion

Present study details the process of immobilization of the enzyme* Candida rugosa *lipase in microemulsion based organogels and its application for synthesis of the flavor ester pentyl valerate in organic solvents. The manuscript mainly focuses on effects of the immobilization and reaction parameters at individual level and at an interactive level. The statistical tool response surface methodology has been employed for study of interactions of the process parameters which (1) reduces the number of experiments and (2) gives a model and prediction of possible outcomes within the given range of parameters.

### 3.1. Effect of Various Parameters on Ester Synthesis

#### 3.1.1. Effect of Surfactant Used for Microemulsion Based Organogel Preparation

**Figure 1:**
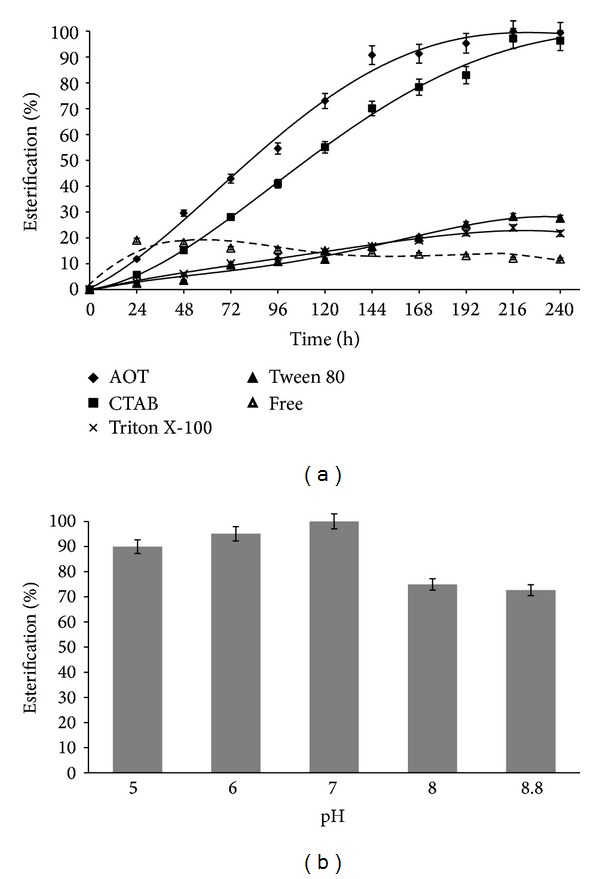
(a) Effect of surfactant on pentyl valerate synthesis. Free enzyme and immobilized enzyme are represented by dotted and solid lines, respectively. The MBGs consisted of surfactant/isooctane/CRL (70 mg/mL), Wo = 60. (b) Effect of pH on pentyl valerate synthesis. CRL was dissolved in the buffers of pH 5, 6, 7, 8, and 8.8 and used for MBG preparation. Organogel system comprised AOT/isooctane, Wo = 60. The reaction was carried out at 37°C and 150 rpm in cyclohexane.

Surfactants are amphiphilic molecules that are used for preparation of reverse micelles containing an aqueous core in a nonaqueous environment. One of the vastly studied surfactants used for microemulsion preparation is AOT [17]. Though organogels have been prepared using various surfactants, AOT has been found to be the surfactant of choice for many researchers [14]. In present study, esterification data revealed that the organogels prepared using AOT/isooctane assisted in highest yield when compared to MBGs prepared using other surfactants (Figure 1(a)). Also, it was observed in our previous work that organogels prepared using nonionic surfactants Triton X-100 and Tween 80 exhibited poor esterification which may be due to their low Wo values (Table S1) and weak and fragile MBGs [16]. CTAB organogels were porous and sturdy [16] and showed higher ester production when compared to those of Tween 80 and Triton X-100 (Figure 1(a)). Similar results were observed by Skagerlind et al. who showed that there was a considerable difference between the reaction rates in AOT and C_12_EO_5_ microemulsion systems which was due to the nonionic surfactant (C_12_EO_5_) competing with the substrate for the active site of lipase [18]. Hence, further experimentation was carried out using AOT based organogels.

#### 3.1.2. Effect of Buffer pH on Ester Synthesis

The fact that enzymes exhibit an optimum pH value and the unusual properties exhibited by the “water pool” of reverse micelles necessitated the study of effect of pH on ester synthesis. In present work, the results showed that the highest ester yield was obtained in MBGs containing enzyme dissolved in buffer of neutral pH (pH 6-7), whereas it started declining as the pH was increased to 8 and 8.8. From Figure 1(b), it can be clearly seen that highest esterification was observed at pH 7 with slightly lower values at pH 6 and pH 5. However, the ester production decreased drastically at pH 8 and 8.8 (Figure 1(b)). Similar observations were made in other studies [16, 19]. Extensive studies on* Candida rugosa *lipase have been performed and it has been reported that the pH optima of CRL are in the range of pH 6.5–7.5 [20]. This shows that the enzyme exhibited similar pH optima before and after immobilization suggesting the immobilization process to be noninfluential on its optimum pH.

#### 3.1.3. Effect of Wo on Esterification

**Figure 2:**
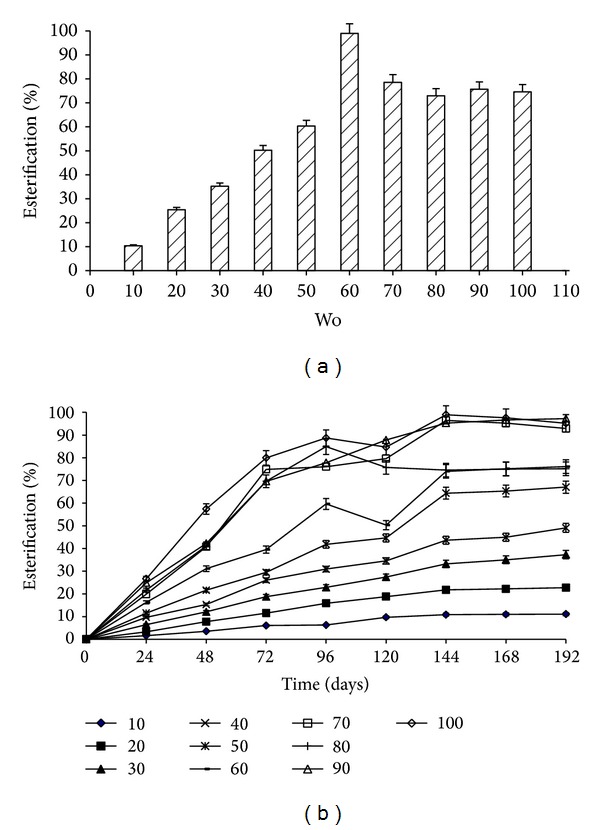
(a) Effect of Wo on pentyl valerate synthesis. Reaction system consisted of AOT/isooctane organogels prepared using varying Wo values and 5 mg/mL of reverse micelles lipase solution. (b) Effect of enzyme concentration on ester synthesis. MBGs containing lipase (10–100 mg/mL) of the combination AOT/isooctane at Wo 60 were prepared. Esterification was carried out at 37°C and 150 rpm in cyclohexane.

The water activity Wo (also known as R sometimes) is one of the most important parameters contributing to the stability of reverse micelles and consequently to organogels prepared using them. Also, the concept of “superactivity” of lipases within reverse micelles makes it an important parameter to be observed. Present study exhibited optimum Wo = 60 wherein 98.93% pentyl valerate was synthesized (Figure 2(a)). Similar result was obtained in our previous study in synthesis of the ester ethyl valerate [16]. In agreement with the results, Stamatis et al. have stated that the typical MBGs used for immobilization of enzymes have a characteristic of high Wo values (Wo > 40) [17]. Also, a similar observation was made by Otero et al. wherein the CRL (isolipase A) exhibited stability at higher Wo values [21].

#### 3.1.4. Effect of Enzyme Concentration on Ester Synthesis

In enzyme catalyzed reactions, there is a strong correlation between the concentration of enzyme and final product yield. In present study, the concentration of enzyme within the reverse micelles was varied while keeping the Wo constant with value of 60. A gradual increase in pentyl valerate synthesis was observed when the concentration of enzyme was increased from 10 mg/mL (11.08%) to 60 mg/mL (76.09%) albeit in a span of 8 days whereas high turnover (96.43%) was observed at 70 mg/mL concentration in a period of 6 days (Figure 2(b)). The increment with further increase in enzyme concentration was not significant. This indicated that, for any given MBG system (with specified Wo value), the ester production increases linearly with increase in concentration up to a certain level. Beyond this optimum concentration, further increment in concentration does not contribute to any significant addition in ester production. More detailed study of interactions is shown in Section 3.2. Similar results have been reported by Monot et al. synthesizing butyl butyrate wherein the esterification increased with increment in concentration from 0.5 gL^−1^ to 2.5 gL^−1^ and decreased when enzyme concentration was increased to 5.0 gL^−^1 [22].

#### 3.1.5. Effect of Organic Solvents as Medium on Ester Synthesis

**Figure 3:**
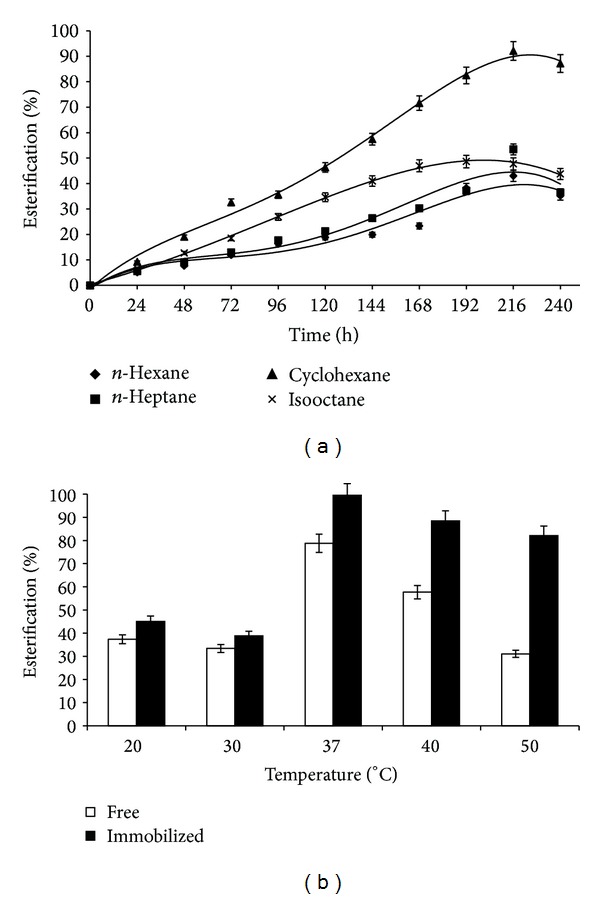
(a) Effect of organic solvents as medium on pentyl valerate synthesis. The reaction system comprised AOT/isooctane organogels (pH 7, Wo = 60, enzyme concentration of 70 mg/mL) in isooctane, n-heptane, n-hexane, and cyclohexane at 37°C and 150 rpm. (b) Effect of reaction temperature on pentyl valerate synthesis. The reaction system comprised AOT/isooctane organogels (pH 7, Wo = 60, enzyme concentration of 70 mg/mL) in cyclohexane.

Nonaqueous biocatalysis has been popularized in the past few decades due to numerous advantages [23]. It has been explained that absence of water molecules from enzymes in anhydrous solvents contributes to a rigid structure due to restrained conformation. This makes them resistant to high temperatures at the cost of lower catalytic activity due to loss of flexibility required for enzyme-substrate interaction. However, this flexibility can be restored strikingly by addition of minute quantities of water [24]. In present work, it can be seen that highest ester production occurred in cyclohexane (92%), followed by n-heptane (53.4%), isooctane (48.63%), and n-hexane (42.99%) (Figure 3(a)). Generally, organic solvents with Log *P* values >2 are found to be excellent candidates for biocatalytic reactions. Laane et al. stated that, among the various properties of solvents, Log *P* is the best to correlate with enzyme activity [25]. However, this study showed that cyclohexane (Log *P* = 3.4) assisted in the highest ester production as compared to n-hexane (Log *P* = 3.5), n-heptane (Log *P* = 4.3), and isooctane (Log *P* = 4.5) indicating that high yields could be obtained in lesser hydrophobic solvents as well (the greater is the Log *P*, the higher is the hydrophobicity of the solvent). Similar results have been reported by Chin et al. [26] and Knubovets et al. [27]. In fact, Castro showed that the enzyme subtilisin exhibited appreciable enzyme activity which was only detected in hydrophilic solvents such as glycerol, ethylene glycol, and 1,3-propanediol [28].

A similar pattern was observed in our previous study [16] regarding cyclohexane exhibiting exceptionally higher turnover when used as reaction medium. The similar trend observed in our previous and present studies initiated the comparative study of n-alkanes, in their straight chain and cyclic forms as reaction medium for ester synthesis using MBGs. It can be seen from Figure S1 that, as expected, higher conversion took place when cyclic alkanes were used than their acyclic counterparts. Similar behavior was observed by Jenta et al. who observed higher activities in presence of branched and cyclic hydrocarbons [29]. Also, it was observed that, with increase in number of carbon in the solvent molecule, there was an increase in ester production. Especially, highest ester product formation was observed in cyclooctane (8C). The 8C hydrocarbons have been reported to assist in high turnovers using MBGs of agar and HPMC [30]. Nascimento et al. also showed that n-octane showed better results for synthesis of n-pentyl oleate catalyzed by* C. viscosum *lipase immobilized in gelatin organogels [31]. Apart from MBGs, cycloalkanes have shown to be better than their acyclic equivalents for esterification in other systems such as nanoconjugates of lipase and multiwalled carbon nanotubes [32].

#### 3.1.6. Effect of Temperature

As temperature is another important physical parameter for biological and chemical reactions, this study was performed in the range of 20–50°C and highest yield (99.52%) was observed at 37°C (Figure 3(b)). The ester yields of free and immobilized lipase were more or less similar at temperatures <37°C which indicated that the enzyme exhibited properties similar to free enzyme even after immobilization. But at temperatures higher than 37°C, immobilized lipase showed higher conversion than that of free form which indicates the resistance offered by the immobilization matrix to the enzyme. Similar results have been reported by Bezbradica et al. and Pereira et al. who have showed similar temperature optima for esterification reactions catalyzed by CRL [33, 34].

#### 3.1.7. Substrate Concentration and Reaction Kinetics

One of the most important aspects of enzyme catalyzed reactions is the study of reaction kinetics. Various workers have studied the reaction kinetics of esterification reactions [35, 36]. In present study, the K_m_ values suggested that both forms of enzyme displayed higher affinity towards pentanol (*K*_*m*(free  enzyme)_ = 23.25 mM and *K*_*m*(immobilized)_ = 74.07 mM) than towards valeric acid (*K*_*m*(free  enzyme)_ = 76.92 mM, *K*_*m*(immobilized)_ = 83.33 mM). This also indicated that the free enzyme showed higher affinity towards the substrates than the immobilized form. Other kinetic studies regarding immobilized lipases have also shown a higher *K*_*m*_ for alcohols than towards acids. For example, in a study using CRL immobilized on polypropylene, the *K*_*m*_ values for butyric acid and sulcatol were 9.41 mmol/L and 47.16 mmol/L, respectively [35]. Likewise, the *V*_max⁡_ values also showed that initial rates catalyzed by free enzyme were higher [*V*_max⁡_ (varying pentanol) = 526.31, *V*_max⁡_ (varying valeric acid = 1250)] than immobilized enzyme [*V*_max⁡_ (varying pentanol) = 166.66, *V*_max⁡_ (varying valeric acid) = 142.85]. This can also be observed from Figure 1(a) where the free form shows higher product formation than all the other MBGs within first 24 hours. Also, in case of free enzyme, *V*_max⁡_ for valeric acid (1250) was far higher than that for pentanol (526.31), whereas the *V*_max⁡_ values for both substrates in immobilized forms were comparable. This implies that, at high concentrations of pentanol, slight inhibition was observed in case of free enzyme whereas immobilization resulted in protection of the enzyme resulting in comparable *V*_max⁡_ value as that obtained for valeric acid. Various studies have been performed on effect of substrate concentration on reaction kinetics of esterification reactions catalyzed by lipases such as* C. viscosum, C. antarctica, *and* M. miehei *lipases immobilized in MBGs [29, 37, 38]. All these studies have shown that although increase in alcohol concentration results in an increase in ester production, higher concentration of alcohol exerts inhibitory effect on the enzyme.

**Figure 4:**
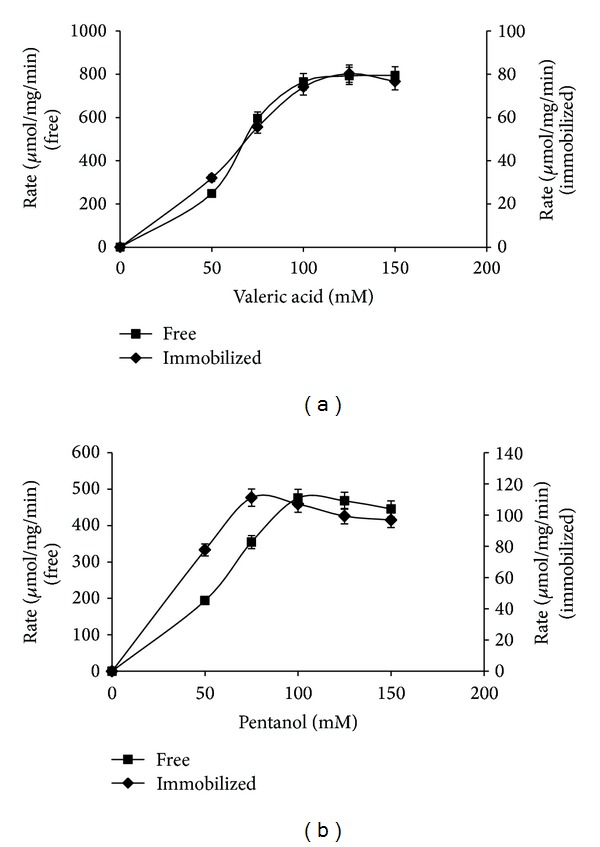
Dependence of initial reaction rate of ester synthesis on concentration of (a) valeric acid concentration (while keeping pentanol concentration constant at 100 mM) and (b) pentanol (while keeping valeric acid concentration constant at 100 mM). The reaction was carried out at 37°C and 150 rpm in cyclohexane as reaction medium.

This was in close agreement with the effects of substrate concentration on reaction rates (Figures 4(a) and 4(b)). High initial rates were observed in case of free enzyme. This can be contributed to the direct contact with the reaction mixture which encourages initial fast reaction whereas, in MBGs, the diffusional limitations slow down the rate. The pattern of substrate saturation was similar for free and immobilized CRL in case of varying valeric acid concentration. However, the dependency curves of pentanol clearly show that, in case of free enzyme, the rate increased initially from 50 to 75 mM alcohol concentration followed by a decrease thereafter indicating inhibition. The immobilized enzyme showed an increase in rate till 100 mM alcohol concentration and stabilized thereafter (Figure 4(b)). Similar observation was made by Varma and Madras during the kinetic study of synthesis of butyl butyrate by Novozyme 435, wherein higher concentrations of alcohol resulted in inhibition while higher concentrations of the acid did not [39]. However, there are other studies which show that there is inhibition by the acid moiety [40] and few more have proved that inhibition can be due to both of the substrates [41].

### 3.2. Study of the Effects of Parameters Using Response Surface Regression Analysis

Response surface methodology (RSM) has been applied for various processes in the field of biocatalysis such as for optimization of enzyme immobilization onto/into supports [42] and study of biocatalyst and reaction parameters for ester synthesis in organic solvents [43–45]. With respect to lipase catalyzed esterification reactions, RSM has been applied for certain systems such as those using free enzyme [46], enzyme immobilized in alginate beads [42], and adsorbed enzyme [47]. However, RSM has not been applied for studying the various interactions of process parameters regarding ester synthesis using lipase immobilized in MBGs. Henceforth, this study was undertaken in present work. The reaction temperature was kept constant at 37°C and cyclohexane was used as reaction medium as they were found to be the optimum conditions in previous sections.

The results of the 45-run BBD of the five variables, namely, enzyme concentration, time, substrate ratio, Wo, and solvent for MBG preparation, are displayed in Table 2. The percentage of esterification varied distinctly in a wide range of 22.49–88.96%. It was observed that high yields were obtained in experimental runs with higher value of the process parameters (+1 coded value). It was also observed that all three solvents used for MBG preparation supported in high ester yields and in most cases, among the three, the organogels prepared using n-hexane showed the best results followed by n-heptane and isooctane. However, it can be seen from Run numbers 42 and 44 wherein, except for the solvent used for MBG preparation, all the rest of parameters were the same (enzyme concentration, time, and substrate ratio at midpoint level and Wo at +1), high (and equivalent) ester yields were obtained. This indicates that all the three solvents were equally efficient for MBG preparation and that the minute differences in the yields can be considered insignificant.

#### 3.2.1. Model Fitting

RSM conjugated 5-factor-3-level BBD was found to be significantly efficient in explaining the interactions of the five process parameters studied and their cumulative effects on ester yield. Blocking was employed to facilitate experimentation and study the variation created (if any) in product yield when the experiments were performed at different points of time. The 45 experiments were run in 5 blocks of 9 experiments each (Table 2) and RSM was applied to fit the second order polynomial equation (4) to the experimental results as shown in Table 2. Among all the experiments, Run numbers 4 (enzyme concentration: 80 mg/mL, time: 9 days, substrate ratio: 1 : 1, Wo: 60, and solvent: n-heptane) and 8 (enzyme concentration: 80 mg/mL, time: 6 days, substrate ratio: 3 : 2, Wo: 60, and solvent: n-heptane) showed highest esterification (88.96% and 88.39%, resp.) and Run number 1 (enzyme concentration: 30 mg/mL, time: 3 days, substrate ratio: 1 : 1, Wo: 60, and solvent: n-heptane) showed lowest esterification (22.49%). It can be observed that, in Run numbers 1 (lowest esterification) and 4 (highest esterification), except enzyme concentration and time, other three parameters were at same level (midpoints). This showed that, even if other parameters are kept at low values, high ester formation can be obtained by keeping enzyme concentration and reaction time at high levels. However, the time duration could be reduced at the cost of increase in substrate concentration, which also yielded 88.39% ester formation (Run number 8). Again, as mentioned in previous section, high yields were also obtained in Run numbers 42 (enzyme concentration: 55 mg/mL, time: 6 days, substrate ratio: 1 : 1, Wo: 80, and solvent: n-hexane) and 44 (enzyme concentration: 55 mg/mL, time: 6 days, substrate ratio: 1 : 1, Wo: 80, and solvent: isooctane) (84.34 and 85.74%, resp.) which implies two things: (1) high ester formation at midpoint values of all parameters except Wo exerting a strong positive effect of Wo and solvent and (2) the effect of solvent used for MBG preparation being very insignificant as both of these runs were carried out using MBGs of different solvents and the results obtained were identical. All these observations clearly indicated that the parameters under study were interacting with each other and are explained in the following section. The fact that high alcohol : acid ratio drove the reaction towards a high product formation (Run number 8, Table 2) shows that pentanol exerted a positive effect on esterification. Zoumpanioti et al. have mentioned in their review that, in comparison to very short chain (1-2C) and long chain (9-10C) alcohols, medium chain (4–6C) alcohols result in high ester yields [14]. The possible reason for this was attributed to the difference in partitioning of alcohols between the solvent used as medium and the microenvironment of the enzyme.

Using the statistical tool, the predicted values were obtained and found to be satisfactorily correlated to observed values (Table 2). Fitting of the data showed that the esterification (%) was best described by the quadric polynomial model as shown in

(5) Y=(26.993)+(12.557x1)+(11.318x2) +(7.390x3)+(7.418x4)−(2.187x5)−(0.859x12) −(2.692x22)−(5.996x32)−(0.521x42)−(1.312x52) +(0.071x1x2)+(4.308x1x3)−(2.124x1x4) −(0.265x1x5)+(3.081x2x3)−(0.866x2x4) +(0.694x2x5)+(2.179x3x4) −(1.23x3x5)+(0.827x2x5).

The regression coefficients of all the terms are shown in Table 3. The single variable coefficients show that all the chosen quantitative factors were highly significant (*P* = 0.000) on esterification (%), with solvent type used for MBG preparation being weakly significant (*P* = 0.033) which was also shown in previous paragraph. The positive sign and high values of coefficients also indicate that higher response would be observed at the +1 value of the variables except for solvent which indicates that esterification would be more at −1 level representing n-hexane. The coefficient of square terms indicates that time and substrate ratio (*X*_2_ and *X*_3_) were highly significant (*P* < 0.05).

**Figure 5:**
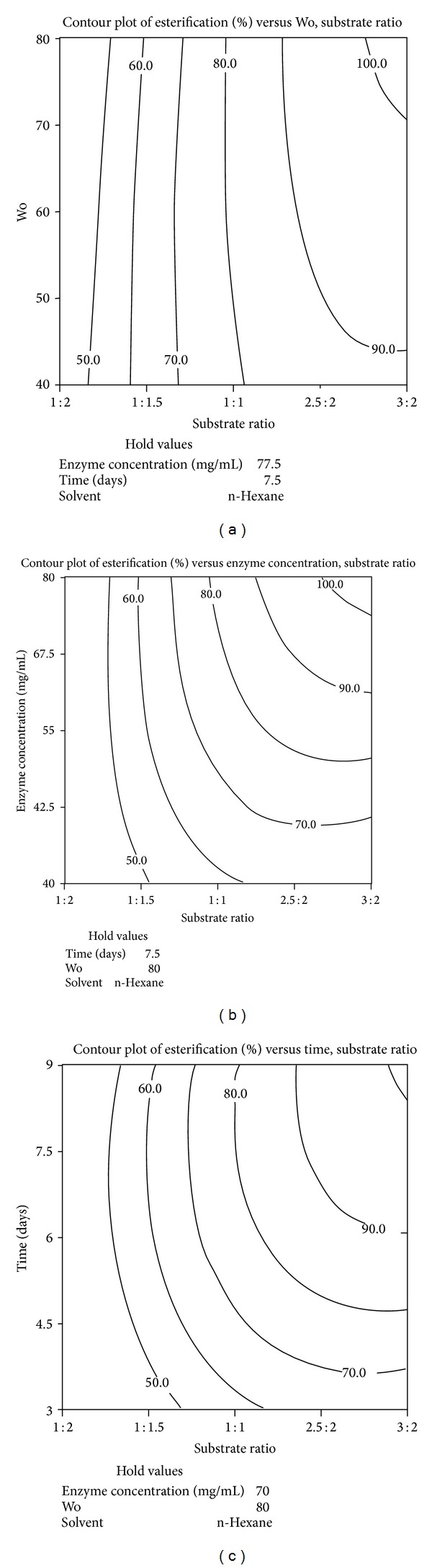
Contour plot for the esterification (%) as a function of (a) substrate ratio and Wo using n-hexane as solvent used for MBG preparation with hold values of time at 7.5 days (coded value 0.5) and enzyme concentration at 77.5 mg/mL (coded value 0.9). (b) Enzyme concentration and substrate ratio using n-hexane as solvent used for MBG preparation with hold values of time at 7.5 days (coded value 0.5) and enzyme concentration at 80 mg/mL (coded value 1). (c) Time and substrate ratio using n-hexane as solvent used for MBG preparation with hold values of Wo = 80 (coded value 1) and enzyme concentration at 70 mg/mL (coded value 0.6).

The coefficients of interactions showed that only four interactions, that is, enzyme concentration-substrate ratio (*P* = 0.000), time-substrate ratio (*P* = 0.003), substrate ratio-Wo (*P* = 0.033), and enzyme concentration-Wo (*P* = 0.038), were significant with respect to the response (Table 3). The signs of the interaction terms' coefficients (− and +) imply that highest response would be obtained if all the quantitative variables are kept at their highest levels (+), while reasonably high esterification (%) is expected at some lower-higher values factorial combinations. This fact is more evident in contour plots shown in Figures 5(a)–5(c).

The analysis of variance (ANOVA) for the model is shown in Table S2. This analysis shows that all the regression terms, that is, linear, square, and interaction, were statistically highly significant (*P* = 0.000). It also showed that the blocks were insignificant (*P* = 0.404) which implies that, even though experiments were carried out in sets, the error was very insignificant to be accounted for. The lack of fit was insignificant and, hence, the second order regression surface model was satisfactorily significant. The coefficient of determination *R* ^2^ = 0.90 shows that the model can explain about 90% of variability and, hence, it is adequate enough to present the true relationship between the variables and the response.

#### 3.2.2. Illustration of Interactive Effects Using Contour plots

Contour plots were prepared for better understanding of the effect of interactions between the variables on the response (esterification). As stated in previous section, the interactions of all parameters (except solvent) with substrate ratio were significant and, hence, these interactions, namely, substrate ratio: Wo, substrate ratio: time, and substrate ratio: enzyme concentration, were chosen as models for elaboration of the cumulative effect of parameters on ester production. n-Hexane based organogels were used for all the above mentioned interactions (Figures 5(a)–5(c)).

Figure 5(a) shows the interactions of the variables substrate ratio and Wo while the other parameters are at hold values of enzyme concentration at 77.5 mg/mL (coded value 0.9), time at 7.5 days (coded value 0.5), and n-hexane (coded value −1) as solvent for MBG preparation. It is clearly visible from the figure that yield as high as 80% ester could be produced at substrate ratio of 1 : 1 (1-pentanol : valeric acid) and any value of Wo between 40 and 80. To increase the turnover further to 90–100% required to raise the levels of both the parameters. This shows that, with an increase in pentanol concentration, higher ester yields could be obtained albeit with increasing the Wo value as well.

Figure 5(b) depicts the interactive effects of substrate ratio and enzyme concentration on esterification at hold values of other parameters: time at 7.5 days (coded value 0.5), Wo at 80 (coded value 1), and n-hexane as solvent (coded value −1). It can be observed from the figure that a product yield of 50% could be obtained at very low enzyme concentrations as well as substrate ratio. To obtain higher ester yield, the values of both parameters need to be increased. Also, a turnover as high as 90% ester could be produced at enzyme concentration of ~61 mg/mL by simply increasing the substrate ratio to the maximum coded level. This implies two things: (1) substrate ratio is one of the key parameters which can decide the fate of ester yield even at lower concentrations of enzyme and (2) the enzyme activity was enhanced by increasing pentanol concentration. Hence, using lower concentrations of enzyme, the system can be tailored to increase the yields by adjusting the initial ratio of substrate concentrations.

Figure 5(c) illustrates the relation between substrate ratio and incubation time with hold values of other parameters at enzyme concentration of 70 mg/mL (coded value 0.6), Wo = 80 (coded value 1), and n-hexane as solvent. The figure displays an interesting interaction between the two process parameters. It shows that yields up to 70–90% can be obtained in lesser number of days (time at lower coded values) by increasing the substrate ratio accordingly. However, to obtain 100% turnover, both parameters had to be increased to their maximum coded levels.

In a nutshell, it is evident from the three contour plots (Figures 5(a)–5(c)) that substrate ratio, enzyme concentration, and Wo exerted strong effects on final ester yields as individual entities and as interactive pairs which is also supported by the values of regression coefficients as shown in Table 3. Their significance as a single factor could be seen at their lower coded values, wherein high response in the form of pentyl valerate could be obtained by adjusting the other parameter being studied.

#### 3.2.3. Validation of the Model

Two confirmatory experiments were carried out using the optimum values of variables for validation of the model. Here, the values of the five variables were taken as follows.

**Experiment 1 .**

Enzyme concentration is of 77.5 mg/mL, time 7 days, 4 h, and 48 min, substrate ratio = 2.8 : 2, Wo = 80, and the solvent n-hexane for MBG preparation. The predicted value was 99.63% and the observed value was 99.87%.

**Experiment 2 .**

Enzyme concentration is of 70 mg/mL, time 8 days, 7 h, and 6 min, substrate ratio= 2.9 : 2, Wo = 80, and n-hexane for MBG preparation. The predicted value was 99.88% and the actual value was 99.95%.

Hence, both of these experiments fully justified the model and showed that it could be applied for pentyl valerate synthesis using MBGs in organic solvents.

### 3.3. Operational Stability of MBGs

**Figure 6:**
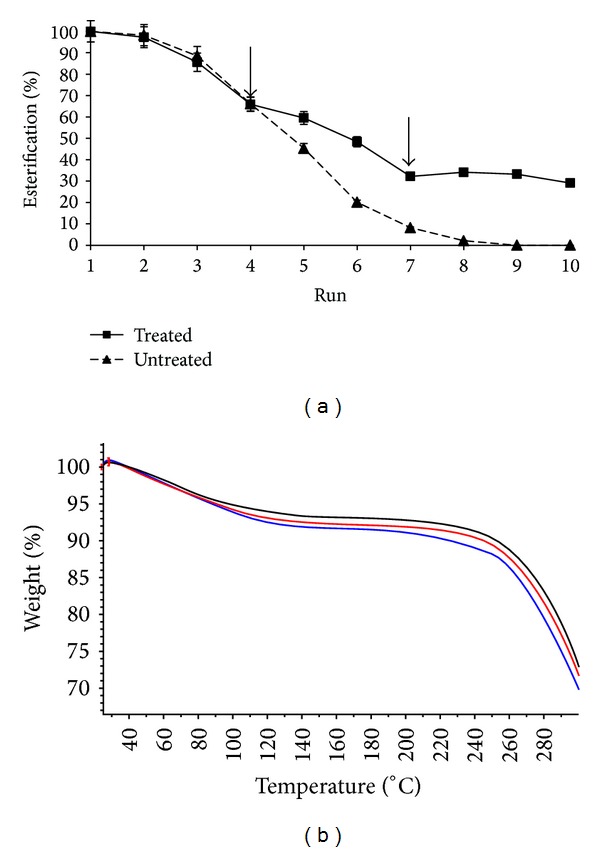
Operational stability of the MBGs. (a) Graph showing number of reusability runs. The organogels were given solvent washes after every run and reused for pentyl valerate synthesis at 37°C and 150 rpm. Down arrows indicate AOT/isooctane treatment given to the organogels for extraction of accumulated water formed as by-product of esterification reaction. (b) TGA weight loss curves for fresh (blue curve), reused (red curve), and reused and treated with 1 M AOT/isooctane (black curve).

The primary aim of immobilization is to use the enzyme repeatedly, apart from easy downstream processing of the product. It was observed that the activity of the enzyme was quite high for first two reaction cycles but decreased gradually thereafter (Figure 6(a)). This could be a consequence of the water that is formed as a by-product of esterification reaction and progressive accumulation in organogels leading to the reverse hydrolysis reaction and partly due to denaturation effects of solvents. To extract this excess water, a treatment of the dehydrant solution, that is, 1 M AOT/isooctane, was given to the used MBGs. This treatment was shown to exert a positive effect on ester synthesis as seen from Figure 6(a). Similar effect was observed after the 6th run. Though a gradual decline in ester yield followed with each repetitive cycle, the extent of this decline was lower than the one displayed by untreated MBGs (Figure 6(a)). Thus, the application of surfactant solution was found to be successful to extract the excess water from organogels leading to restoration of the equilibrium favored towards synthetic reaction as seen in retention of ester produced in the 8th run. Similar observations have been made by Dandavate and Madamwar while studying synthesis of ethyl isovalerate using surfactant coated* Candida rugosa *lipase immobilized in MBGs [48].

Further, to confirm the action of AOT/isooctane solution for extracting excess water, the three types of MBGs were analyzed by TGA weight loss (Figure 6(b)) and Karl-Fischer titration (Table S3) for determining their water content. Karl-Fischer titration results showed that the fresh MBG contained 10.86 ± 0.9% water corresponding to the Wo (= 60) whereas, in the reused MBGs (without treatment), the water content increased by almost 1.5-fold (14.65 ± 2%). The organogels treated with 1 M AOT/isooctane displayed a significant extraction of water showing 8.23 ± 3% water content by Karl-Fischer analysis. This also showed that the treated MBGs possessed slightly lower water content than the fresh MBGs indicating a possible extraction of the original water in addition to the excess by-product. From the TGA weight loss curves, it can be seen that the weight loss in fresh and reused MBGs (without treatment) was much faster as compared to the weight loss observed for the reused and treated MBG (Figure 6(b)). Though the trend of weight loss curves was similar for all the three MBGs, there was a speedy loss observed for reused (without treatment) MBG till around 90–100°C as compared to the other two organogels. The loss in this range of temperature was also comparatively high for fresh organogel. This can be contributed to the water (including accumulated water in case of used MBG) which evaporates by 100°C resulting in a faster loss of weight.

Hence, regarding the reusability studies, two important findings were observed. Firstly, there is an accumulation of water within the MBGs which acts as the substrate and hence drives the equilibrium towards the hydrolysis resulting in reduced ester formation. This excess water can be extracted by a simple treatment by incubating with a dehydrant solution such as AOT/solvent. Secondly, the reduced ester formation after 3-4 runs of esterification is mainly contributed to the shifting of equilibrium towards hydrolysis due to water accumulation (and by a small factor due to enzyme denaturation) which can be restored by a large factor using dehydrant solution.

These two findings support the fact that indeed immobilization of enzymes within MBGs not only facilitates easy separation and renders them reusable, but also offers protection to a great extent and simple methods of recovery of enzyme activity. Our previous studies have shown that the enzyme within MBGs can resist high temperatures up to 60°C for 10 h and 70°C for 4 h indicating the thermostability offered by immobilization matrix [16]. Studies by Backlund et al. [49], Jenta et al. [50], and Rees et al. [51] have shown that immobilization of enzymes within MBGs renders the enzyme highly stable and assists in retaining their activities to a very high extent. Present study also demonstrated that indeed this method of immobilization combats harmful effects of organic solvents and supports the enzyme to be productive under extreme conditions.

## 4. Conclusions 

Present study outlines the synthesis of the flavor ester pentyl valerate using* Candida rugosa *lipase in free as well as in immobilized forms. Immobilization of enzyme within MBGs aided in 2.5–5-fold increase in ester synthesis compared to free enzyme. Organogels prepared using AOT and cycloalkanes as reaction medium were best suited for ester synthesis. Kinetic studies showed that CRL exhibited higher affinity towards pentanol than valeric acid, and the immobilized enzyme exhibited enhanced activity at higher concentrations of pentanol. Scrutiny of five parameters at individual and combinatorial levels using RSM studies revealed their strong interactive effects on ester synthesis. A second order equation was deduced which could be utilized for prediction of ester formation using the known values of the five factors studied herein. Two validation experiments were performed yielding ~100% pentyl valerate production. The immobilized enzyme was used for 10 cycles with appreciable productivity and intermittent AOT/isooctane treatment was found to be an effective method for extraction of excess water from MBGs which was confirmed by Karl-Fischer titration and TGA.

## Supplementary Material

In Table S1, the Wo and Po values are important parameters describing the reverse micelles structure. Wo refers to the ratio of concentration of water to that of concentration of surfactant (*[*water*]* : *[*surfactant*]*) and Po refers to the ratio of concentration of cosurfactant to that of concentration of surfactant (*[*cosurfactant*]* : *[*surfactant*]*). Present results showed that reverse micelles prepared using AOT and CTAB displayed the highest Wo values using all the three solvents used.
In Table S2, ANOVA or analysis of variance shows that all the regression terms, that is, linear, square, and interaction, were found to be statistically highly significant (*P*=0.000). The lack of fit was insignificant and, hence, the second order regression surface model was satisfactorily significant. The coefficient of determination was *R^2^* = 0.90 indicating that the model can explain about 90% of variability and, hence, it is adequate enough to present the true relationship between the variables and the response.
In Table S3, the MBGs containing lipase were applied for catalyzing esterification reaction in organic solvents. Water is formed as a byproduct of esterification reaction and accumulates within MBGs. After few cycles of reaction, significant amount of water is accumulated within MBGs resulting in the slowing down of ester formation. Hence, this excess water was extracted using a dehydrant solution, that is, 1M AOT/isooctane. The reused, untreated, and treated MBGs were analyzed using Karl-Fischer analysis and TGA for water content. Table S2 displays that the AOT/isooctane treatment was effective in extracting excess water from reused MBGs.
In Figure S1, straight chain n-alkanes and their cyclic counterparts were compared as medium for esterification reaction using lipase immobilized in MBGs. The figure shows that each cyclic alkane assisted in higher ester production when compared to the corresponding straight chain alkane.

## Figures and Tables

**Table 1:** Experimental range and coded values of the variables.

Process variables	Coded values		
	−1	0	+1
Enzyme concentration (mg/mL) (*X*_1_)	30	55	80
Time (days) (*X*_2_)	3	6	9
Substrate molar ratio (*X*_3_)	1 : 2	1 : 1	3 : 2
Wo (*X*_4_)	40	60	80
Solvent for MBG preparation (*X*_5_)	n-Hexane	n-Heptane	Isooctane

**Table 2:** Full factorial Box-Behnken design for synthesis of pentyl valerate using MBGs in cyclohexane.

Block	Number	Enzyme concentration	Time	Substrate ratio	Wo	Solvent	Actual (%)	Predicted (%)
1	1	−1	−1	0	0	0	22.49	22.99
	2	1	−1	0	0	0	53.41	55.58
	3	−1	1	0	0	0	53.29	52.90
	4	1	1	0	0	0	**88.96**	86.25
	5	−1	0	−1	0	0	41.83	32.72
	6	1	0	−1	0	0	42.81	43.90
	7	−1	0	1	0	0	36.88	31.12
	8	1	0	1	0	0	**88.39**	87.78
	9	0	0	0	0	0	66.18	65.66

2	10	−1	0	0	−1	0	31.97	29.45
	11	1	0	0	−1	0	80.18	72.64
	12	−1	0	0	1	0	54.05	58.89
	13	1	0	0	1	0	78.15	82.89
	14	−1	0	0	0	−1	38.09	42.96
	15	1	0	0	0	−1	82.51	79.75
	16	−1	0	0	0	1	32.97	38.30
	17	1	0	0	0	1	71.86	72.25
	18	0	0	0	0	0	63.8	66.73

3	19	0	−1	−1	0	0	30.3	20.85
	20	0	1	−1	0	0	36.08	34.65
	21	0	−1	1	0	0	27.31	25.19
	22	0	1	1	0	0	70.85	71.96
	23	0	−1	0	−1	0	24.16	21.67
	24	0	1	0	−1	0	61.08	56.58
	25	0	−1	0	1	0	41.48	46.14
	26	0	1	0	1	0	71.83	71.79
	27	0	0	0	0	0	62.5	59.52

4	28	0	−1	0	0	−1	40.27	41.17
	29	0	1	0	0	−1	67.36	67.74
	30	0	−1	0	0	1	32.92	31.38
	31	0	1	0	0	1	68.21	64.01
	32	0	0	−1	−1	0	33.38	33.85
	33	0	0	1	−1	0	41.13	43.02
	34	0	0	−1	1	0	38.11	42.04
	35	0	0	1	1	0	73.67	74.53
	36	0	0	0	0	0	61.87	61.68

5	37	0	0	−1	0	−1	41.11	41.37
	38	0	0	1	0	1	59.57	56.45
	39	0	0	−1	0	1	37.76	42.85
	40	0	0	1	0	1	55.77	56.45
	41	0	0	0	−1	−1	57.6	60.57
	42	0	0	0	1	−1	**84.34**	75.99
	43	0	0	0	−1	1	41.33	49.72
	44	0	0	0	1	1	**85.74**	74.00
	45	0	0	0	0	0	64.48	69.86

**Table 3:** Estimated regression coefficients for esterification (%) for enzyme concentration (*X*_1_), incubation time (*X*_2_), substrate ratio (*X*_3_), Wo (*X*_4_), and solvent for MBG preparation (*X*_5_).

Term	Coefficient	Standard error coefficient (SE)	*T*	*P*
*X*_ 1_	16.7997	1.338	12.557	**0.000**
*X*_ 2_	15.1419	1.338	11.318	**0.000**
*X*_ 3_	10.4122	1.409	7.390	**0.000**
*X*_ 4_	9.9234	1.338	7.418	**0.000**
*X*_ 5_	−3.0408	1.390	−2.187	**0.033**
*X*_1_^2^	−3.7261	4.338	−0.859	0.394
*X*_2_^2^	−7.8221	2.906	−2.692	**0.009**
*X*_3_^2^	−13.5324	2.257	−5.996	**0.000**
*X*_4_^2^	−1.1522	2.209	−0.521	0.604
*X*_5_^2^	−3.8029	2.898	−1.312	0.194
*X*_ 1_ *X*_ 2_	0.1887	2.676	0.071	0.944
*X*_ 1_ *X*_ 3_	11.5275	2.676	4.308	**0.000**
*X*_ 1_ *X*_ 4_	−5.6825	2.676	−2.124	**0.038**
*X*_ 1_ *X*_ 5_	−0.7100	2.676	−0.265	0.792
*X*_ 2_ *X*_ 3_	8.2425	2.676	3.081	**0.003**
*X*_ 2_ *X*_ 4_	−2.3162	2.676	−0.866	0.390
*X*_ 2_ *X*_ 5_	1.8575	2.676	0.694	0.490
*X*_ 3_ *X*_ 4_	5.8313	2.676	2.179	**0.033**
*X*_ 3_ *X*_ 5_	−3.7794	3.072	−1.230	0.223
*X*_ 4_ *X*_ 5_	2.2137	2.676	0.827	0.411
