# Supplementary material for: Biocatalytic Synthesis of Flavor Ester “Pentyl Valerate” Using Candida rugosa Lipase Immobilized in Microemulsion Based Organogels: Effect of Parameters and Reusability
Source: Biomed Res Int. 2014 Jul 1;2014:353845. doi: 10.1155/2014/353845 (PMC4100389; doi:10.1155/2014/353845)
Supplement: Supplementary file 1 — In Table S1, the Wo and Po values are important parameters describing the reverse micelles structure. Wo refers to the ratio of concentration of water to that of concentration of surfactant ([water] : [surfactant]) and Po refers to the ratio of concentration of cosurfactant to that of concentration of surfactant ([cosurfactant] : [surfactant]). Present results showed that reverse micelles prepared using AOT and CTAB displayed the highest Wo values using all the three solvents used. In Table S2, ANOVA or analysis of variance shows that all the regression terms, that is, linear, square, and interaction, were found to be statistically highly significant (P=0.000). The lack of fit was insignificant and, hence, the second order regression surface model was satisfactorily significant. The coefficient of determination was R2 = 0.90 indicating that the model can explain about 90% of variability and, hence, it is adequate enough to present the true relationship between the variables and the response. In Table S3, the MBGs containing lipase were applied for catalyzing esterification reaction in organic solvents. Water is formed as a byproduct of esterification reaction and accumulates within MBGs. After few cycles of reaction, significant amount of water is accumulated within MBGs resulting in the slowing down of ester formation. Hence, this excess water was extracted using a dehydrant solution, that is, 1M AOT/isooctane. The reused, untreated, and treated MBGs were analyzed using Karl-Fischer analysis and TGA for water content. Table S2 displays that the AOT/isooctane treatment was effective in extracting excess water from reused MBGs. In Figure S1, straight chain n-alkanes and their cyclic counterparts were compared as medium for esterification reaction using lipase immobilized in MBGs. The figure shows that each cyclic alkane assisted in higher ester production when compared to the corresponding straight chain alkane. [file 353845.f1.pdf]

Supplementary information

Biocatalytic synthesis of flavor ester 'pentyl valerate' using lipase immobilized in microemulsion based organogels: Impact of parameters on ester production

Tripti Raghavendra[1], Nilam Panchal[1], Jyoti Divecha[2], Amita Shah[1], Datta Madamwar*[1]

1. BRD School of Biosciences, Sardar Patel Maidan, Satellite Campus, Vadtal Road, Post Box No. 39, Sardar Patel University, Vallabh Vidyanagar – 388 120, Gujarat, India

2. Department of Statistics, Sardar Patel University, Vallabh Vidyanagar – 388 120, Gujarat, India

* Corresponding author:

Tel: +91 2692 229380

Fax: +91 2692 231042

E-mail address: datta_madamwar@yahoo.com (D. Madamwar)

raghavendra.tripti@gmail.com (T. Raghavendra)

Table S1: Wo and Po values of the reverse micellar systems prepared using the four surfactants AOT, CTAB, Tween 80 and Triton X-100 in combination with the three organic solvents n-hexane, n-heptane and isooctane. 1-hexanol was used as the co-surfactant in case of CTAB, Tween 80 and Triton X-100.

**Surfactant**	**Organic solvents**					
	**Isooctane**		**n-hexane**		**n-heptane**	
	Wo	Po	Wo	Po	Wo	Po
**AOT**	60	-	50	-	70	-
**CTAB**	30	14.43	60	12.83	80	9.62
**Triton X 100**	50	15.92	30	14.43	50	19.24
**Tween 80**	40	12.83	40	11.22	50	16.04

Table S2: The analysis of variance (ANOVA) for esterification (%)

Source	Degree of freedom	Sum of squares	Mean of squares	F value	P value
Blocks	9	2702.1	60.81	1.06	0.404
Regression	20	28242.2	1412.11	24.66	**0.000**
Linear	5	22895.2	4541.32	79.29	**0.000**
Square	5	3009.4	524.09	9.15	**0.000**
Interaction	10	2337.6	233.76	4.08	**0.000**
Residual error	60	3436.4	57.27	-	-
Lack of fit	58	3406.5	58.73	3.92	0.224
Pure error	20	29.9	14.97	-	-
Total	89	34380.7	-	-	-

$\boldsymbol{R^2}$ **= 0.90** $\boldsymbol{R^2_{adj}}$**= 0.85**

Table S3: Estimation of residual water using Karl-Fischer titration and TGA weight loss curves

**Organogel**	**Karl-Fischer titration**	**TGA weight loss analysis**	
	**Water (%)**	**Residual weight (%)**	**Water (%)**
**Fresh**	10.86 ± 0.9	88.16 ± 2	11.84
**Reused**	14.65 ± 2	87.75 ± 0.9	12.25
**Reused and treated with AOT/isooctane**	8.23 ± 3	89.27± 2	10.73

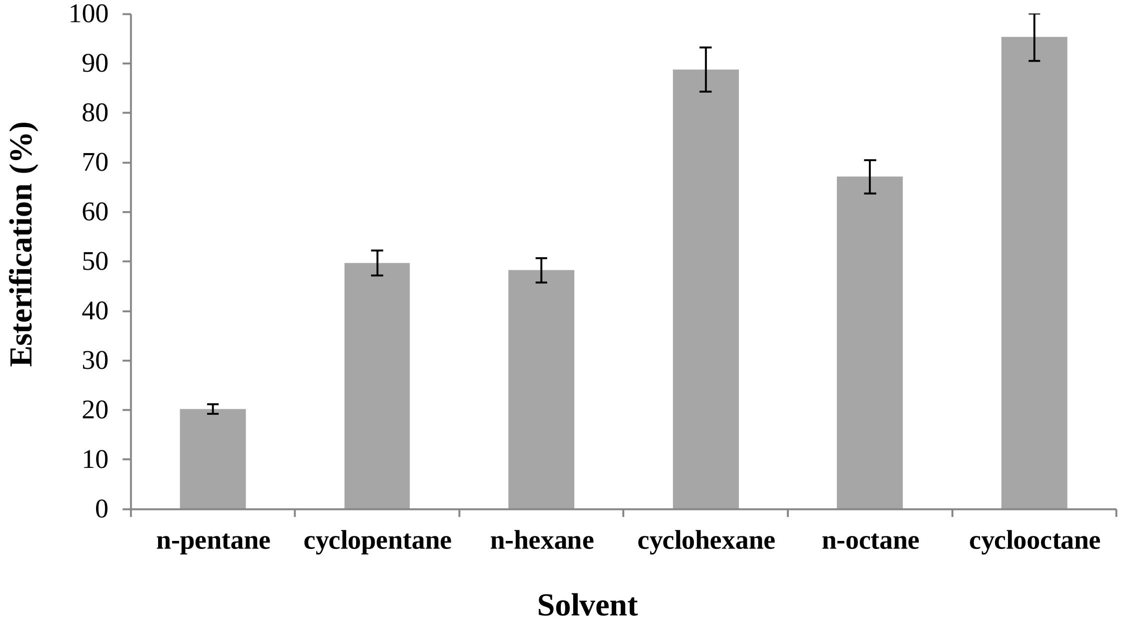

Figure S1: Effect of straight chain and cyclic hydrocarbons on ester synthesis. The reaction was carried out using AOT/isooctane/gelatin MBGs at 37 °C and 150 rpm for 96h.
